# The Nutritional Value and Health Benefits of Chickpeas and Hummus

**DOI:** 10.3390/nu8120766

**Published:** 2016-11-29

**Authors:** Taylor C. Wallace, Robert Murray, Kathleen M. Zelman

**Affiliations:** 1 Department of Nutrition and Food Studies, George Mason University, Fairfax, VA 22030, USA; 2 Department of Human Sciences, The Ohio State University, Columbus, OH 43210, USA; murraymd@live.com; 3 Atlanta Nutrition Communications, Atlanta, GA 30062, USA; kzelman@webmd.net

**Keywords:** hummus, chickpeas, legumes, beans, vegetables

## Abstract

The 2015–2020 Dietary Guidelines for Americans advocate for increasing vegetable intake and replacing energy-dense foods with those that are nutrient-dense. Most Americans do not eat enough vegetables, and particularly legumes, each day, despite their well-established benefits for health. Traditional hummus is a nutrient-dense dip or spread made from cooked, mashed chickpeas, blended with tahini, olive oil, lemon juice, and spices. Consumers of chickpeas and/or hummus have been shown to have higher nutrient intakes of dietary fiber, polyunsaturated fatty acids, vitamin A, vitamin E, vitamin C, folate, magnesium, potassium, and iron as compared to non-consumers. Hummus consumers have also been shown to have higher Healthy Eating Index 2005 (HEI-2005) scores. This may be, in part, due to hummus’ higher Naturally Nutrient Rich (NNR) score as compared to other dips and spreads. Emerging research suggests that chickpeas and hummus may play a beneficial role in weight management and glucose and insulin regulation, as well as have a positive impact on some markers of cardiovascular disease (CVD). Raw or cooked chickpeas and hummus also contain dietary bioactives such as phytic acid, sterols, tannins, carotenoids, and other polyphenols such as isoflavones, whose benefits may extend beyond basic nutrition requirements of humans. With chickpeas as its primary ingredient, hummus—and especially when paired with vegetables and/or whole grains—is a nutritious way for Americans to obtain their recommended servings of legumes. This manuscript reviews the nutritional value and health benefits of chickpeas and hummus and explores how these foods may help improve the nutrient profiles of meals.

## 1. Introduction

The 2015–2020 Dietary Guidelines for Americans (DGA) advocate for healthy eating patterns that include a variety of vegetables from all five of the following vegetable subgroups: dark green, red and orange, legumes (beans and peas), starchy, and other [1]. This includes all fresh, frozen, canned, and dried options in either their cooked and/or raw forms. The recommended intake of vegetables in the Healthy U.S.-Style Eating Pattern at the 2000-calorie level is 2.5 cup-equivalents of vegetables per day. Increasing the amount and variety of vegetables Americans consume is one strategy that helps to ensure Americans meet a wide range of nutrient requirements. However, about three-fourths of the population has an eating pattern that is low in vegetables, fruits, dairy, and oils; on average, no age or gender group, except males and females 1–3 years, meets the 2015–2020 DGA’s recommendations for legume intake [1]. Legumes (i.e., beans and peas) include kidney beans, pinto beans, white beans, black beans, garbanzo beans (chickpeas), lima beans (mature, dried), split peas, lentils, and edamame (green soybeans). The USDA Economic Research Service estimates that only 14% of Americans consume dried beans on a semi-regular basis (note: this number does not account for canned beans) [2]. Since legumes have a similar nutrient profile to both vegetables and protein foods, they may often be used to fulfill requirements of both food groups. Legumes are known as an excellent source of dietary protein. While the proteins present in legumes are not considered “complete”, as compared to most animal-derived proteins, when combined with foods such as whole grains (e.g., whole grain bread), a balanced intake of essential amino acids can be easily achieved. Heat treatment also significantly improves the protein quality of legumes, such as chickpeas, since it destroys and/or inactivates many heat liable anti-nutritional factors [3,4,5]. This may be of increasing importance for vegans and individuals adhering to variations of plant-based diets.

Chickpeas (*Cicer arietinum* L.), commonly known as garbanzo beans, are an old world pulse (i.e., edible seeds) in the legume family [1] and have traditionally been incorporated into many culinary creations because of their nut-like flavor and versatile sensory applications in food [3]. The origin of the chickpeas is thought to have been Levant and ancient Egypt [6], which is logical since the plant prefers temperate and semiarid regions. India is the world’s leading producer of chickpeas. Worldwide over 14.2 metric tons of chickpeas were harvested in 2014 according to the Food and Agriculture Organization (FAO) of the United Nations [7]. Two main varieties of chickpeas exist: the light seeded Kabuli type and the smaller dark Desi type [8]. Pulses are unique in comparison to other plant foods in that they contain higher proportions of protein (17%–30% by dry weight). The main proteins found in chickpeas, similar to other legumes, are albumins and globulins. Smaller amounts of glutelins and prolamines are also present [9].

In Western culture, chickpea consumption is somewhat driven through the intake of hummus. Traditional hummus is a dip or spread made from cooked, mashed chickpeas, blended with tahini, olive oil, lemon juice, and spices. A variety of other forms of hummus—or bean-based dips labeled as hummus that do not follow the traditional hummus recipe—exist on the market, each containing unique ingredients which may or may not contribute to nutrient intakes and/or have benefits beyond basic nutrition. Data in the peer-reviewed literature and summarized in this review predominantly pertains to the traditional hummus recipe. It should be noted that, while chickpeas and hummus share a similar nutrient profile, they are often not nutritionally equivalent (Table 1). For instance, commercial processing of hummus may alter the profile and bioavailability of some nutrients. Over the past decade, the hummus market has grown to over $530M in U.S. sales alone as of 2013, increasing by over 25% since 2010 [10]. This could be in part due to increased consumer recognition of pulses (e.g., chickpeas) and their derived products [8] or, in scientific terms, their high nutritional quality. Hummus, because of its chickpea content, is a source not only of protein, but also dietary fiber, resistant starch, polyunsaturated fatty acids, vitamins, and minerals, especially folate, calcium, magnesium, and potassium (Table 1). Four tablespoons (~100 kcal) of traditional, chickpea-based hummus per day provides approximately 2 cups of legumes per week and ~25 grams of dietary fiber—a shortfall nutrient in the diets of many adults and children. Raw or cooked chickpeas and hummus also contain dietary bioactives such as phytic acid, sterols, tannins, carotenoids, and other polyphenols such as isoflavones, whose benefits may extend beyond the basic nutrition requirements of humans [11]. This manuscript seeks to review the scientific literature in regard to nutritional aspects of chickpeas and hummus as part of a healthy dietary pattern.

## 2. Chickpea and Hummus Consumers, Compared to Non-Consumers, Have Been Suggested as Having Improved Nutrient Intakes and Diet Quality

Chickpeas and/or hummus supports the healthy eating patterns recommended by both the Mediterranean-style diet and the 2015–2020 DGA. Chickpea and hummus consumers have been shown to have better HEI-2005 subcomponent scores for total vegetables, dark green and orange vegetables, and whole grains, as compared to non-consumers [12]. The 2015–2020 DGA propose that individuals should strive to consume vegetables (including legumes) in a nutrient-dense form with limited additions of salt, butter, or creamy sauces [1]. Traditionally prepared hummus may act as a healthier, tasty alternative to the many dips and spreads traditionally served. Observational studies suggest that chickpea, pulse, and legume consumption helps to increase intakes of several essential nutrients. Recent analysis of adult participants (≥19 years) enrolled in the National Health and Nutrition Examination Survey (NHANES) show that those who consume chickpeas/hummus have higher intakes of dietary fiber (24.4 ± 0.7 vs. 10.1 ± 0.1 g/day), polyunsaturated fatty acids (19.5 ± 0.4 vs. 17.3 ± 0.1 g/day), vitamin A (787 ± 42 vs. 640 ± 6 RAE mcg/day), vitamin E (10.1 ± 0.5 vs. 7.5 ± 1.01 mcg/day), vitamin C (119 ± 8 vs. 86.4 ± 4.3 mg/day), folate (627 ± 16 vs. 547 ± 4 mcg/day), magnesium (385 ± 13 vs. 292 ± 4 mg/day), potassium 3103 ± 59 vs. 2697 ± 12 mg/day), and iron (17.4 ± 0.5 vs. 15.8 ± 0.1 mg/day), as compared to non-consumers. Consumers also had lower intakes of total fat (76.4 ± 4.5 vs. 80.4 ± 0.3 g/day), saturated fat (22.4 ± 0.7 vs. 26.6 ± 0.1 g/day), and cholesterol (227 ± 8 vs. 288 ± 2 mg/day). Consumers also had lower intakes of total fat (76.4 ± 4.5 vs. 80.4 ± 0.3 g/day), saturated fat (22.4 ± 0.7 vs. 26.6 ± 0.1 g/day), and cholesterol (227 ± 8 vs. 288 ± 2 mg/day). Chickpea/hummus consumers had higher Healthy Eating Index (HEI)-2005 scores, as compared to non-consumers (62.2 ± 1.2 vs. 51.9 ± 0.2) [12]. Similarly, other studies have also shown bean consumption (not specific to chickpeas) to be associated with higher nutrient intakes, particularly two nutrients of public health concern (potassium and dietary fiber) and shortfall nutrients for some populations including vitamin A, vitamin E, vitamin C, folate, iron, and magnesium [13], as outlined by the 2015–2020 DGA [1]. Mitchell et al. also found regular consumption of pulses (half a cup per day) to result in higher-quality diets, including higher intakes of dietary fiber, protein, folate, zinc, iron, and magnesium, and lower intakes of saturated fat and total fat [14]. Feeding studies also support an increase in dietary fiber intake with the consumption of chickpeas [15,16].

## 3. Hummus, Compared to Other Dips and Spreads, Has Greater Nutrient Density

Nutrient density is defined as the ratio of the nutrient composition of a food to the nutrient requirements of a human. The 2015–2020 DGA recommend replacing some foods with more nutrient-dense options [1]; however, “nutrient-dense” foods lack a common definition [17]. The Naturally Nutrient Rich (NNR) score is a universally accepted nutrient-to-calorie ratio that can be calculated as the average of the percent daily value (%DV) for 16 nutrients: NNR = Σ%DV_2000Kcal_/16 [18,19]. Using data from the U.S. Department of Agriculture National Nutrient Database, the nutritional profile and NNR scores were calculated for common dips and spreads (Table 2). Traditional hummus may help consumers maximize their nutrient-to-calorie ratio when choosing a dip or spread because of its unique blend of health promoting ingredients. While counting calories has been a traditional strategy for weight control [20], application of this nutrient density standard helps consumers make decisions that maximize each calorie towards achieving intake recommendations.

## 4. Health Outcomes Associated with Consumption of Chickpeas and Hummus

Traditional hummus contains a unique combination of chickpeas, tahini, olive oil, lemon juice, and spices that may provide additional benefits beyond satisfying nutrient requirements. While the scientific literature is emerging, several studies support hummus/chickpea consumption in relation to weight control, glucose, and insulin response, cardiovascular disease, cancer, and/or GI health.

### 4.1. Weight Control

In general, diets high in fiber, low in energy density and glycemic load, and moderate in protein are thought to be particularly important for weight control [21]. In the NHANES 2003–2010 dataset, chickpea/hummus consumers were 53% less likely to be obese and 51% less likely to have an elevated glucose level. Likewise, consumers had a lower body mass index (BMI) (26.4 ± 0.5 vs. 28.6 ± 0.1) and waist circumference (92.2 ± 1.3 vs. 97.9 ± 0.3 cm) compared to non-consumers [12]. This could be somewhat due to other healthy lifestyle patterns that one might expect individuals that have higher intakes of pulses such as chickpeas to exhibit (NHANES is observational data and cannot assess causality). Pulse consumption, alone [13] or included in a dietary pattern [22,23,24], has also been associated in epidemiologic studies with reduced body weight, waist circumference, and risk of overweight and obesity. Consumption of chickpeas/hummus has additionally been suggested as affecting markers of both metabolic syndrome and cardiovascular disease in both human and animal intervention studies (discussed below).

### 4.2. Glucose and Insulin Response

Chickpeas have a low glycemic index [11,15,25,26]; however, very few studies have assessed the glycemic effects of hummus in vivo. Post-prandial glucose responses were four times lower than that of white bread in a study of 10 healthy subjects consuming hummus. Blood glucose levels were significantly lower after 45 minutes when subjects were fed hummus with 25 g of available carbohydrates (in the form of white bread), as compared to 25 g of carbohydrates alone (note: no differences in blood glucose levels were found at time intervals prior to 45 min post-consumption). This suggests that hummus may be able to partially attenuate the effects of foods with a higher glycemic index upon consumption [25]. When consumed long-term, chickpea intake also significantly improved glycemic control in a 20-week crossover study of 45 individuals with elevated cardiovascular disease (CVD) risk factors [15]. Other human intervention studies have shown pulses to lower glycemic responses by slowing the rate of carbohydrate absorption when compared to an isoglucidic standard [26,27]. Mollard et al. showed blood glucose response to be dependent on pulse type in 25 young healthy males. Men who consumed either chickpeas or lentils with a pizza meal had lower blood glucose responses as compared to those who consumed yellow peas with a pizza meal [28]. Cross-sectional analysis of NHANES 2003–2010 did not show any cross-sectional association with fasting insulin or glucose levels in those who reported consuming chickpea/hummus intake vs. non-consumers [12]. Yang et al. showed that chickpeas significantly improve insulin resistance and prevent post-prandial hyperglycemia and hyperinsulinemia induced by a chronic high-fat diet in rats [29]. Likewise, emerging epidemiological evidence shows that pulse consumption is associated with a decreased risk for type-2 diabetes [30,31].

Traditional hummus has a fat content 4-5 times that of chickpeas alone (Table 1), which may account for the improved blood glucose and insulin response, since dietary fat delays gastric emptying and therefore slows carbohydrate absorption [32,33]. The glycemic index of hummus is approximately half that of chickpeas [34], which could partially explain why larger decreases in blood glucose levels in the study with hummus vs. those with chickpeas were shown. While blood glucose and insulin response may be attenuated by hummus consumption, it should be noted that the additional fat content contributes additional calories to the diet.

### 4.3. Cardiovascular Disease

A controlled dietary intervention study suggested that isoenergetic chickpea supplementation of a wheat-based Australian-style diet brought about significant reductions in serum total cholesterol (TC) and low-density lipoprotein cholesterol (LDL-C) [35]. Similarly, chickpea intake significantly improved TC and LDL-C control in a 20-week crossover study of individuals with elevated CVD risk factors [15]. Analysis of NHANES did not show any cross-sectional association with fasting lipid profiles, blood pressure, or C-reactive protein (CRP) levels in those who reported consuming chickpea/hummus intake vs. non-consumers [12]. A recent meta-analysis of randomized controlled trials indicated that a pulse-rich diet decreases LDL-C [36]. Systolic blood pressure decreased in overweight and obese individuals after pulse consumption for eight weeks [28]. The activities of lipoprotein lipase in the epididymil adipose tissue and hepatic triacylglycerol lipase in the liver were normalized in the rats on a high-fat plus chickpea diet, which is likely somewhat related to lower levels of leptin and lipoprotein lipase mRNA content reported in the epididymil adipose tissue [25]. In a mechanistic animal study, rats fed a high fat plus chickpea diet also showed less visceral adiposity and improved lipid profiles after eight months as compared to those on a high fat diet alone [29].

Soluble fiber is well known for its positive effects on TC and LDL-C, which are recognized validated biomarkers of cardiovascular disease [37]. Some studies have also shown an increased benefit of increased vegetable protein intake in relation to CVD [38]. The higher amounts of dietary fiber and protein content of chickpeas, and possibly the presence of enzyme inhibitors and “antinutrients” such as tannins present in chickpeas, may also help partially explain these findings.

### 4.4. Cancer

Butyrate is a principal short chain fatty acid (about 18% of the total volatile fatty acids) produced from the consumption of a chickpea diet (200 g/day) in healthy adults [39]. Butyrate has been widely reported to suppress cell proliferation [40] and induce apoptosis [41], which may reduce the risk of colorectal cancers. Several other dietary bioactive compounds, such as lycopene, Biochanin A, and saponins that have been shown to reduce the risk of certain types of cancers are also present in chickpeas and hummus [11]. Murillo et al. showed a 64% suppression of azoxymethane-induced aberrant cryptic foci in rats supplemented with 10% chickpea flour. This study suggested that a high concentration of saponins in the chickpea flour could partially account for the reported reductions in lesions [42]. Similarly, the inclusion of chickpea seed coat fiber in the diet has been shown to decrease the toxic effects of *N*-nitrosodiethylamine on lipid peroxidation and antioxidant potential [43]. While there is no data specific to hummus in this area, an epidemiological study found that pulse consumption was also associated with a reduced risk of some cancers [44].

### 4.5. Gastronintestinal Tract Health

Dietary fiber is the indigestible part of plant foods, containing poly/oligosaccharides, lignin, and other plant substances. Dietary fiber is classified into soluble and soluble fibers. Soluble fibers are slowly digested in the colon, while insoluble fibers are indigestible and promote bowel movements. Similar to other plant foods, legumes, and pulses, significant increases in dietary fiber intake have been reported when chickpeas and/or hummus is added to the diet [12,16,45]. Human studies of chickpeas report overall improvements in bowel health characterized by increased frequency of defecation, ease of defecation, and softer stool consistency while on a chickpea diet as compared to a habitual diet [16,45].

## 5. Hummus: Opportunity to the Improve Nutritional Profile of Meals and to Improve the Intake of Other Vegetables and Whole Grains

Menu modeling analysis (MMA) is a technique used by nutrition and health professionals to optimize a restaurant menu or a diet’s nutritional profile. MMA reinforces that hummus is a nutrient-dense way to help support decreased calorie intakes from saturated fats and refined carbohydrates. Data from NHANES 2003–2010 suggests that consumers of hummus have significantly higher HEI 2005 subcomponent scores for total vegetables (3.8 ± 0.1 vs. 3.1 ± 0.02), dark green and orange vegetables (2.7 ± 0.1 vs. 1.3 ± 0.02), and whole grains (1.8 ± 0.1 vs. 1.1 ± 0.02) [12]. The traditional hummus-improved menu examples provided (Table 3) show improvements in meeting protein or vegetable (legume) recommendations as set by the 2015–2020 DGA. Substituting other dips, spreads, and ingredients with hummus illustrates measurable improvements in the nutritional profile of meals. Figure 2-2 in the 2015–2020 DGA illustrates hummus with carrots as a healthy shift from a high-calorie snack (i.e., potato chips) to one that is more nutrient dense [1]. The hummus-substituted menu examples provided (Table 3) have the potential to provide an average reduction in total kcal (−6%), total fat (−16%), saturated fat (−28%), sodium (12%), and sugars (23%). These improvements are attributed to the substitution of hummus for other, more energy-dense spreads, dips, and ingredients. For example, substituting 1 Tbsp. of butter for hummus on a baked potato saves approximately 65 kcal.

## 6. Conclusions

Chickpeas and hummus are an easy means to help consumers meet the recommended 1.5 cups of legumes per week. Four tablespoons (~100 kcal) of traditional, chickpea-based hummus per day provides approximately 2 cups of legumes per week and ~25 grams of dietary fiber—a shortfall nutrient in the diets of many adults and children. This same amount also provides approximately 14 g of plant protein per week, as well as many other essential vitamins and minerals. Substitution of common dips and spreads with hummus helps to increase diet quality. Finally, emerging evidence suggests that chickpea and hummus consumption has benefits beyond providing basic nutrition. Consuming chickpeas and/or hummus may help prevent or offset the development and progression of several chronic diseases (CVD, type-2 diabetes, etc.) and promote healthier functional outcomes (e.g., weight management). Consuming chickpeas and/or hummus in moderation may have additional benefits beyond improving nutrient profiles of meals (e.g., delaying gastric emptying and slowing carbohydrate absorption); however, more clinical research is needed across various subpopulations.

## Figures and Tables

**Table 1:** Nutritional profile of chickpeas and hummus.

Nutrient	Unit	DV ^b^	Value per 100 g ^a^		
			Chickpeas, Dry (16056) ^c^	Chickpeas, Cooked (16057) ^c^	Hummus (16158) ^c^
Macronutrients					
Energy	Kcal	2000	378	164	166
Protein	g	50	20.47	8.86	7.90
Fat	g	78	6.04	2.59	9.60
Carbohydrate	g	275	62.95	27.42	14.29
Fiber	g	28	12.2	7.6	6.0
Sugar	g		10.7	4.8	NR
Minerals					
Calcium	mg	1300	57	49	38
Iron	mg	18	4.31	2.89	2.44
Magnesium	mg	400	79	48	71
Phosphorus	mg	1000	252	168	176
Potassium	mg	4700	718	291	228
Sodium	mg	2300	24	7	379
Zinc	mg	15	2.76	1.53	1.83
Copper	mg	2	0.656	0.352	0.527
Manganese	mg	2	21.306	1.030	0.773
Selenium	μg	70	0	3.7	2.6
Vitamins					
Vitamin C	mg	60	4.0	1.3	0
Thiamin	mg	1.5	0.477	0.116	0.180
Riboflavin	mg	1.7	0.212	0.063	0.064
Niacin	mg	20	1.541	0.526	0.582
Pantothenic acid	mg	10	1.588	0.286	0.132
Vitamin B6	mg	2	0.535	0.139	0.200
Folate	μg	400	557	172	83
Choline	mg	550	99.3	42.8	NR
Vitamin B12	μg	6	0	0	0
Vitamin A	IU	5000	67	27	30
Vitamin D	μg	20	0	0	0
Vitamin K	μg	80	9.0	4.0	NR
Vitamin E	mg	30	0.82	0.35	NR
Lipids					
Saturated	g	20	0.603	0.269	1.437
Monounsaturated	g	ND	1.377	0.583	4.039
Polyunsaturated	g	ND	2.731	1.156	3.613

^a^ Data obtained from the USDA National Nutrient Database for Standard Reference; ^b^ Based on a caloric intake of 2000 kcal, for adults and children four of more years of age; ^c^ Nutrient Database Number (NDB No.) in the USDA Food Composition Databases. (https://ndb.nal.usda.gov/ndb/). DV = daily value; NR = not reported; ND = no data.

**Table 2:** Nutritional profile and NNR scores of hummus and other common spreads and dips.

	Hummus (16158)	Bean Dip (27065)	Ranch Dressing (04639)	Salsa (06164)	Sour Cream (01056)	Cream Cheese (01017)	Peanut Butter (16098)
Energy (kcal) ^a^	50	43	129	10	48	102	191
Total Fat (g) ^a^	2.88	1.33	13.36	0.06	4.64	9.99	16.44
Saturated Fat (g) ^a^	0.431	0.190	2.089	0.008	2.434		3.304
Carbohydrates (g) ^a^	4.29	5.72	1.77	2.39	1.11	1.60	7.14
Fiber (g) ^a^	1.8	1.8	0.0	0.7	0.0	0.0	1.6
Protein (g) ^a^	2.37	1.96	0.40	0.55	0.59	1.78	7.11
Sodium (mg) ^a^	114	159	270	256	7	91	136
**NNR Score**	**98.42**	**82.36**	**23.02**	**89.29**	**42.95**	**41.86**	**67.94**

^a^ Data obtained from the USDA National Nutrient Database for Standard Reference. Note: Amounts listed are per 2 tbsp. serving. NNR = Naturally nutrient rich.

**Table 3:** Menu modeling examples of dietary improvements by substituting other dips, spreads, and ingredients with hummus.

**Adults**	
Menu 1	Hummus substituted for mayonnaise in potato salad
	Hummus and baby carrots substituted for potato chips and French onion dip
Menu 2	Hummus on whole wheat toast substituted for wheat toast with butter and jelly
	Hummus substituted mayonnaise and ketchup on a hamburger and French fries
Menu 3	Tuna salad prepared with hummus and served on a whole grain pita substituted tuna salad prepared with mayonnaise on a croissant
	Hummus and celery substituted a pudding snack
Menu 4	Hummus substituted for bacon on a breakfast burrito
	Hummus substituted for butter on a baked potato
Menu 5	Mediterranean pizza with hummus, chicken and vegetables substituted a cheese and sausage pizza
	Hummus substituted for mashed potatoes as a side dish
**Children**	
Menu 1	Pasta tossed with hummus substituted macaroni and cheese
	Hummus with whole grain crackers substituted an applesauce cup
Menu 2	Hummus substituted ranch dressing as a dip for vegetables
	Hummus substituted mayonnaise on a turkey sandwich
